# Memory and belief updating following complete and partial reminders of fake news

**DOI:** 10.1186/s41235-024-00546-w

**Published:** 2024-05-07

**Authors:** Paige L. Kemp, Alyssa H. Sinclair, R. Alison Adcock, Christopher N. Wahlheim

**Affiliations:** 1 Department of Psychology, University of North Carolina at Greensboro, 296 Eberhart Building, P. O. Box 26170, Greensboro, NC 27402-6170 USA; 2 Department of Psychology and Neuroscience, Duke University, Durham, NC 27708 USA; 3 Center for Science, Sustainability, and the Media, University of Pennsylvania, Philadelphia, USA; 4 Department of Psychiatry and Behavioral Sciences, Duke University, Durham, USA

**Keywords:** Corrections, Fake news, Familiarity backfire, Integration, Misinformation

## Abstract

Fake news can have enduring effects on memory and beliefs. An ongoing theoretical debate has investigated whether corrections (fact-checks) should include reminders of fake news. The familiarity backfire account proposes that reminders hinder correction (increasing interference), whereas integration-based accounts argue that reminders facilitate correction (promoting memory integration). In three experiments, we examined how different types of corrections influenced memory for and belief in news headlines. In the exposure phase, participants viewed real and fake news headlines. In the correction phase, participants viewed reminders of fake news that either reiterated the false details (complete) or prompted recall of missing false details (partial); reminders were followed by fact-checked headlines correcting the false details. Both reminder types led to proactive interference in memory for corrected details, but complete reminders produced less interference than partial reminders (Experiment 1). However, when participants had fewer initial exposures to fake news and experienced a delay between exposure and correction, this effect was reversed; partial reminders led to proactive facilitation, enhancing correction (Experiment 2). This effect occurred regardless of the delay before correction (Experiment 3), suggesting that the effects of partial reminders depend on the number of prior fake news exposures. In all experiments, memory and perceived accuracy were better when fake news and corrections were recollected, implicating a critical role for integrative encoding. Overall, we show that when memories of fake news are weak or less accessible, partial reminders are more effective for correction; when memories of fake news are stronger or more accessible, complete reminders are preferable.

**Supplementary Information:**

The online version contains supplementary material available at 10.1186/s41235-024-00546-w.

Fake news headlines on the internet present verifiably false information as true. Although fake news has long existed, it has recently resurged and influenced attitudes about global events like the 2016 and 2020 US Presidential elections, UK Brexit Referendum, and COVID-19 pandemic (Pennycook & Rand, 2021). Fake news exposure can affect behavior, such as when a fake story about a faulty COVID-19 contact-tracing app reduced download intentions (Greene & Murphy, 2021). Such negative consequences make it imperative to find effective correction strategies. Conflicting evidence has spurred debate about whether corrections should include reminders of misinformation. Reminders can sometimes backfire by increasing familiarity and misperceptions of accuracy (Autry & Duarte, 2021). However, such backfire has not always been observed (Prike et al., 2023). Reminders can also diminish misinformation’s influence on memory, reasoning, and beliefs (Ecker et al., 2017). The effects of these reminders depend on study designs and stimuli (Swire-Thompson et al., 2020, 2022; Wood & Porter, 2019) as well as the extent that reminders promote remembering that misinformation was corrected (Kemp et al., 2022a; Wahlheim et al., 2020). The available evidence therefore suggests that reminders of fake news may improve or impair correction, crucially depending on whether and how false details are reinstated, as well as how fake news and corrections are linked in memory.

Misinformation continues to influence memory, reasoning, and beliefs, even after corrections. In early demonstrations of the *continued influence effect*, participants read a story describing an event, read corrections of misinformation from the story, and made inferences about the event (Johnson & Seifert, 1994; Wilkes & Leatherbarrow, 1988). Corrections have consistently reduced but not eliminated the influence of misinformation on inferences when compared with no-correction (misinformation only) and no-misinformation (correction only) controls (for reviews and a meta-analysis, see Lewandowsky et al., 2012; Walter & Tukachinsky, 2020). This has been observed widely in real-world claims (Lewandowsky et al., 2005) and urban myths (Swire et al., 2017), as well as consumer and social media behaviors (MacFarlane et al., 2021; Tay et al., 2022). Different accounts offer competing predictions about how reminders of misinformation should impact correction efficacy.

The *selective retrieval* account posits that the continued influence effect occurs when misinformation is automatically activated (Ecker et al., 2010; Gordon et al., 2019), such as when misinformation is more accessible than correct information, or when source monitoring fails (Jacoby, 1991; Yonelinas, 2002). Accordingly, repeating misinformation before a correction can exacerbate the continued influence effect (Ecker et al., 2011) because reminders increase misinformation familiarity and thus perceived accuracy (Pennycook et al., 2018; Schwarz et al., 2007; Unkelbach, 2007). An extreme version of this view proposes that repeating misinformation induces a *familiarity backfire* effect, increasing perceived accuracy instead of decreasing it (Schwarz et al., 2007), but there is sparse evidence for this familiarity backfire effect on misinformation beliefs (for a review, see Swire-Thompson et al., 2020).

By contrast, the *integration* account proposes that the continued influence effect occurs when memories of misinformation and corrections are not effectively associated during encoding (Ecker et al., 2017; Kendeou et al., 2014, 2019). This view aligns with the idea that reminders can reduce proactive interference from prior experiences by promoting cross-episode associations that support recollection-based retrieval of the relationship between true and false information (Wahlheim et al., 2021). By this view, the continued influence effect occurs when people do not detect the conflict between true and false details, or when there is insufficient co-activation of true and false details to build associative links. Accordingly, corrections with misinformation reminders should promote the co-activation necessary to detect conflict and support integrative encoding. Consistent with this prediction, misinformation reminders have been shown to reduce the continued influence effect more than corrections without misinformation details (Ecker et al., 2017).

The accounts above suggest that reminders can be harmful or helpful when correcting misinformation (for a review, see Ecker et al., 2022). Several factors—including encoding strength, context similarity, and the nature of the retrieval cue—may determine whether co-activation leads misinformation to impair or improve memory for the details of corrections. As memory is one basis for beliefs (Berinsky, 2017; Kowalski & Taylor, 2017; Newman et al., 2022), it is important to investigate how reminders of fake news influence both memory and perceived accuracy. Indeed, misinformation may differentially influence the various stages of persuasion, including recall, knowledge, and behavior (McGuire, 1968, for similar views, see Newman et al., 2022; Walter & Tukachinsky, 2020). Here, we examined how different types of fake news reminders affect both memory for and beliefs in news headlines.

The present study is motivated by investigations of how reminders of fake news headlines, presented before real news, affect memory and belief accuracy (e.g., Wahlheim et al., 2020). In phase 1 of these tasks, participants first viewed real and fake news headlines of unclear veracity. In phase 2, participants viewed real news headlines that corrected fake news or affirmed real news, and real news headlines that only appeared in phase 2 (controls). At test, participants recalled real news details from phase 2, rated their belief that the recalled details were true, indicated if headlines had corrected fake news from phase 1, and if so, recalled the fake news from phase 1. In the initial study, memory and belief accuracy when recalling real news details was highest when fake news reminders preceded real news headlines that corrected the fake news. Fake news reminders also led to better recollection that fake news was corrected and of the fake news itself. Memory for and belief in real news were enhanced when participants recollected corrections and impaired when they did not recollect corrections. A follow-up study showed that reminder benefits exceeded those conferred by labeling corrections, suggesting that reminders had benefits above and beyond simply highlighting conflict salience (Kemp et al., 2022a). Overall, including fake news reminders during corrections with real news details conferred a net benefit by promoting recollection of corrections.

The tacit assumption of these studies showing reminder benefits was that repeating fake news headlines often triggered *recognition* of prior experiences, and thus promoted integrative encoding (cf. Wahlheim et al., 2019). However, the integrative encoding account predicts that corrections that cue *recall* of fake news details should also be effective. A related study examined this possibility using a variant of the fake news correction paradigm (Kemp et al., 2022b). When participants read real news headlines in phase 2, they also indicated which headlines corrected fake news from phase 1 and recalled those fake news details. After detecting corrections and recalling fake news, subsequent recall of real news was enhanced when participants recollected the correction and impaired when they did not.

Collectively, these studies of fake news reminders suggest that reminder-cued recognition and recall can both improve correction efficacy by promoting integrative encoding. However, it is unclear whether these retrieval types confer differential benefits. Unlike recognition, recalling fake news requires self-generation of contextual information, which may lead to differences in the strength and nature of evoked memory representations, thus affecting integrative encoding. This idea that the type of reminder determines the fate of a reactivated memory aligns with evidence from the cognitive neuroscience of memory updating. One account referred to as the *non-monotonic plasticity hypothesis* proposes that memory updating depends on how effectively a reminder reactivates a memory (Ritvo et al., 2019). Accordingly, strong reactivation (due to a strong reminder or strong prior encoding) promotes integration, linking the reactivated memory with new information. Moderately strong reactivation instead leads to differentiation, weakening the prior memory while encoding the new information. Weak reactivation (e.g., due to weak initial encoding or ineffective retrieval) fails to modify the prior memory. Relatedly, other evidence suggests that partial reminders (e.g., probing recall) are crucial for updating memories, perhaps because they facilitate recall processes and elicit surprise (Sinclair & Barense, 2019).

Together, these studies suggest that the type of memory retrieval evoked by different reminders (recognition vs. cued recall) and the efficacy of initial encoding will interact to determine memory updating that supports beliefs in real and fake news. We propose that when people strongly encode misinformation (e.g., repeatedly encountering fake news), *complete reminders* that probe recognition may enhance memory updating more effectively than *partial reminders* that probe recall. This prediction is grounded in the notion that strong reactivation (combining strong encoding with a complete reminder) promotes integrative encoding. However, when people weakly encode misinformation (e.g., one prior exposure), complete reminders may not reactivate the memory strongly enough to promote integration. Thus, the optimal reminder for corrections will depend partly on initial encoding efficacy.

## The present study

The literatures above collectively suggest that memory and belief updating should depend on how misinformation is encoded and subsequently retrieved prior to correction. We investigated this issue in the present three experiments by manipulating the types of retrieval cued by reminders and varying the initial encoding strength of misinformation. This approach allowed us to address the theoretical issue of how reminders that probe recognition and recall affect integrative encoding and subsequent recollection of cross-episode associations and belief updating. The present experiments also offer practical implications for correcting fake news in real-world settings, informing correction methods about how to remind people of fake news based on the frequency of and time since exposure to misinformation.

We based the present paradigms on the three-phase procedures used in the studies of fake news corrections above (Kemp et al., 2022a, b; Wahlheim et al., 2020). Here, participants rated the accuracy of real and fake news headlines of unclear veracity (phase 1), then read real news headlines that corrected fake news and affirmed real news from phase 1 (phase 2), and, finally, completed a cued recall test of real and fake details that also measured memory for corrections (phase 3). Before most real news headlines in phase 2, participants saw *complete reminders* that repeated headlines from phase 1 or *partial reminders* that repeated headlines from phase 1 without the critical detail that could have been fake news. We instructed participants to make recognition judgments for complete reminders and to recall details for partial reminders. We examined the interaction of reminder type with initial encoding by varying the frequency and timing of headlines in phase 1 within and across experiments. To examine general reminder effects, we included a control condition: Participants saw real news headlines in phase 2 that did not correspond to headlines in phase 1 and therefore did not follow reminders. Because belief updating may depend on memory for prior experiences (Newman et al., 2022), and because few studies have considered how corrections affect memory (Kemp et al., 2022a, b; Wahlheim et al., 2020), we focus on memory updating in all experiments and the interaction of memory and belief updating in Experiments 2 and 3.

## Experiment 1

Experiment 1 compared the effects of presenting complete and partial fake news reminders before real news on memory for headline details. Based on related findings (Kemp et al., 2022a, b; Wahlheim et al., 2020), we predicted that memory accuracy would be greater to the extent that reminders promote retrieval of fake news and associative encoding with real news that supports recollection-based retrieval. We expected complete reminders to cue fake news retrieval better than partial reminders, because complete reminders fully reinstate details. Importantly, the familiarity backfire and integrative encoding views make competing predictions about whether the retrieval types, perhaps due to the different demands they place on self-generated context reinstatement, will lead to differences in correction effects.

According to the familiarity backfire view, complete reminders should increase fake news familiarity more than partial reminders, because complete reminders fully reinstate the false details and probe recognition. As a result, complete reminders should lead to more potential for proactive interference relative to partial reminders. Conversely, according to the integrative encoding view, complete reminders should strengthen the associative links between fake news and corrections, leading to better subsequent recollection of details and their veracity following complete reminders relative to partial reminders. Finally, neither account clearly predicts whether the association between the accurate reminder retrieval and subsequent memory should vary between recognition and recall. We explored this relationship by examining final real and fake news recall conditioned on whether fake news details were retrieved earlier during reminders.

### Experiment 1: method

#### Transparency and openness

We report how we determined sample sizes, all data exclusions, all manipulations, and all measures. The deidentified data upon which the study conclusions are based, the code necessary to reproduce the analyses, and the study materials are available on the Open Science Framework (OSF) at https://osf.io/pes2y/. The present research was conducted in compliance with the Institutional Review Boards at Duke University (Protocol #2022–0105) and UNC Greensboro (Protocol #FY22-245).

#### Participants

Our stopping rule was to acquire usable data from at least 60 participants by testing all available participants in one semester. We based the sample size on a sensitivity analysis of the smallest effect size of interest from a study of fake news reminders described above (Wahlheim et al., 2020). We report the sensitivity analysis in the Supplementary Information (henceforth, Additional file 1: SI Section 1). Ninety-three Duke University students (61 women, 28 men, 1 other, and 3 unidentified) ages 18–23 (*M* = 19.20, *SD* = 1.20) participated for course credit.

#### Design

**Fig. 1:**
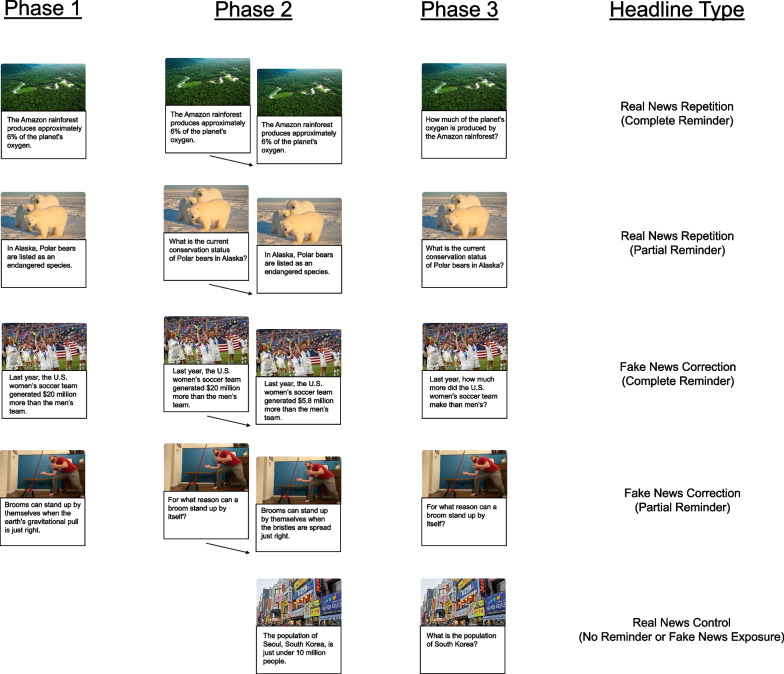
*Example Statements from Each Phase of the Three Experiments.* The trial structures for all within-subjects conditions appear above. The complete set of headlines are available on the OSF (https://doi.org/10.17605/Osf.Io/Pes2y)

**Fig. 2:**
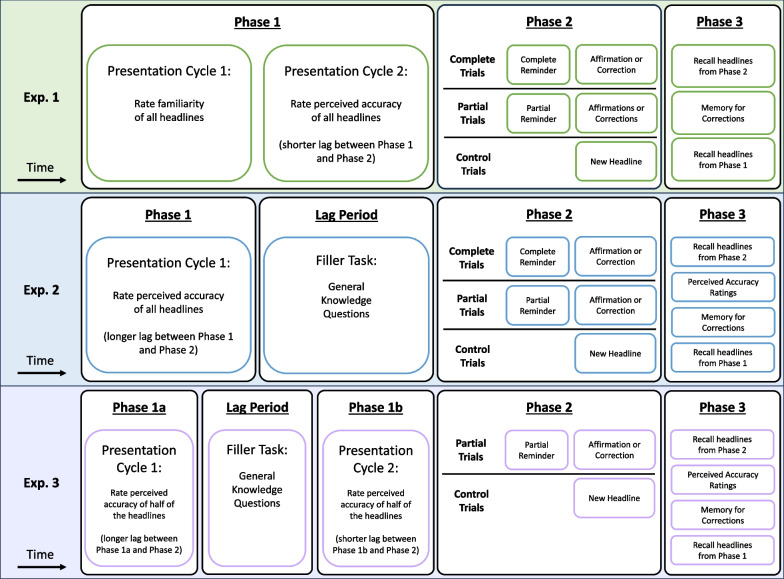
*Schematic of the Procedure*. Phase 1 differed across the three experiments in the following ways. In Experiment 1, participants were exposed to real and fake headlines twice and rated them for familiarity and then perceived accuracy. In Experiment 2, participants were exposed to real and fake headlines once and rated them for perceived accuracy prior to engaging in a distractor task. In Experiment 3, participants rated their perceived accuracy of half of the real and fake headlines (shorter lag) in phase 1a, engaged in a distractor task, and then rated the other half of the real and fake headlines (longer lag) in phase 1b. Another difference across experiments was the reminder types in phase 2. In Experiments 1 and 2, participants saw reminders that either fully reinstated headline details (complete) or prompted participants to recall omitted details (partial) from the phase 1 headlines. In Experiment 3, participants only saw partial reminders. In all three experiments, following a reminder, participants saw real news headlines that affirmed real news form phase 1, corrected fake news, or appeared for the first time as control items without prior fake news exposure. The final difference among experiments was the trial structure in phase 3. In Experiment 1, participants first recalled phase 2 real news details, then indicated whether a correction occurred in phase 2, and for those, attempted to recall fake news from phase 1. In Experiments 2 and 3, participants also attempted to rate the perceived accuracy of details they reported when recalling real news

We included five within-subjects conditions, with four of those conditions emerging from a 2 × 2 crossed factorial design. We manipulated the types of headlines by repeating real news headlines (Real News Repetitions) or correcting fake news headlines (Fake News Corrections) from phase 1 to phase 2. We also manipulated the types of reminders that appeared in phase 2 by repeating phase 1 headlines completely (Complete Reminders) or with a missing detail (Partial Reminders). Finally, we included a control condition with real news headlines that appeared without corresponding fake news in phase 1 or a reminder in phase 2. Figure 1 shows example headlines and presentation formats for all conditions, and Fig. 2 (top panel, Experiment 1) displays a schematic of the procedure.

#### Materials

We obtained news headlines from fact-checking websites (i.e., FactCheck.org, PolitiFact.com, and Snopes.com). All fake news headlines were initially portrayed by the media as being true (i.e., we used actual “fake news” items that were published and later corrected). We created a display format that resembled news updates on internet search engines (e.g., Google). Real and fake news headline details about the same topic appeared in the same font below an image related to the topic. We paraphrased the original headlines to include identical fake and real headline prose except for the fake detail and real detail that corrected it. The set included 70 pairs of real and fake news headlines. We counterbalanced headline assignment by dividing the set into five groups of 14 pairs and rotating groups through conditions, thus producing five experimental formats. Each group included comparable topic varieties, and some topics were deemed more political (i.e., climate change, healthcare, foreign trade) than others (i.e., women’s soccer, removable car headrests, toilet germs), as well as qualitative and quantitative corrections (see Additional file 1, SI Section 2). The complete set of headlines are available on the OSF (https://doi.org/10.17605/Osf.Io/Pes2y).

#### Procedure

Participants completed the task in an internet browser on their personal computers (mobile devices were not allowed), outside of the laboratory, and unsupervised. We instructed them to complete the experiment in a quiet, distraction-free setting in one sitting. The task was programmed with PsychoPy v.2022.2.5 (Peirce et al., 2019) and was hosted by Pavlovia, the PsychoPy companion platform for online data collection. The task comprised three phases and took approximately one hour to complete. Before beginning each phase of the task, participants were required to read the instructions and answer a comprehension check question correctly.

In phase 1, participants first read a series of headlines that included both fake and real news items, rating each headline for familiarity and perceived accuracy. In phase 2, we reminded participants of each of the phase 1 news headlines by presenting either a *complete reminder* (recognition probes that reproduced the full headline) or a *partial reminder* (cued recall probes that omitted a critical detail). After responding to each retrieval cue, participants viewed a “fact-checked” headline that either affirmed the real news or corrected the fake news. In a control condition, previously unseen real news headlines appeared in phase 2, without a reminder. In phase 3, participants completed a cued recall test that assessed memory for real news, fake news, and memory for corrections (i.e., whether fake news had been corrected). The memory for corrections measure provided information about memory for the relationship between real and fake details (i.e., that real news appeared later, correcting fake news).

The phase 1 instructions told participants that their tasks would be to rate the familiarity and accuracy of news headlines across two cycles and to study the headlines for a test. These ratings provided baseline measures and kept participants engaged. In each cycle of phase 1, participants viewed 56 unique headlines (28 real and 28 fake) divided evenly between the reminder-type conditions (14 per condition). All headlines appeared once in cycle 1 before repeating in cycle 2. Headlines appeared individually for 8 s each in random order. While each headline appeared, participants made ratings of familiarity (cycle 1) and accuracy (cycle 2) on four-point scales ranging from 1 (Extremely Unfamiliar) to 4 (Extremely Familiar) and 1 (Definitely False) to 4 (Definitely True). The instructions told participants to use the full rating scale and respond using the corresponding number keys. When participants did not make a rating while the headline appeared, a message appeared for 1.5 s prompting faster responses. The interstimulus interval (ISI) was self-paced; participants advanced trials by clicking a button on the screen.

The phase 2 instructions told participants that their tasks would be to answer questions about their memory for headlines from phase 1 and to study fact-checked verified real news headlines that followed those questions for a future test. The instructions also stated that sometimes the fact-checked verified headline would correct the fake news headline from phase 1, affirm the real news headline from phase 1, or be entirely new (control condition). When fake news headlines were corrected with real news, participants were asked to mentally note the discrepancies to improve their memory for the real news (i.e., to encourage associative encoding). Participants viewed 70 headline topics including the 56 phase 1 topics (14 per reminder condition) and 14 new topics in the control condition. On reminder trials, the real or fake news reminder appeared first, just before the real news headline for that topic. Complete reminder headlines appeared as in phase 1; participants indicated their memory for each complete reminder on a three-point scale from 1 (Do Not Remember) to 3 (Completely Remember). Partial reminder headlines appeared as in phase 1, missing the criterial detail that could have been real or fake; participants recalled missing details by typing their responses. All reminders appeared for 8 s (0.5 s ISI) each, while participants made their responses. The headlines following reminders or appearing alone as control items also appeared for 8 s (0.5 s ISI) each. Headline topics appeared in random order. Participants advanced trials by clicking a button on the screen.

The phase 3 instructions told participants that their task would be to answer memory questions about the headlines they had just studied in phase 2. Participants completed a cued recall test including 70 questions asking about the key detail of each studied headline that could have been corrected. Cues appeared individually in random order with the original image above the question and a text box in which participants typed their responses. On each trial, participants first attempted to recall the real news detail. A prompt then asked participants to indicate whether the phase 2 detail they typed had corrected fake news from phase 1. They responded using the mouse to click on boxes labeled “Yes” and “No.” When participants responded “Yes,” a prompt asked them to type the fake news detail from phase 1. When they responded “No,” the program advanced to the next trial. Participants were encouraged to respond accurately and were allowed to pass when they could not think of a response. After each question, participants used the mouse to click a button to advance.

After phase 3, participants completed a demographic questionnaire that also included a question about their partisanship and a question about their subjective memory ability relative to their age group. We did not have any specific interest concerning the latter two questions and therefore do not report analyses of those data here. The data from these questions are on the OSF (https://doi.org/10.17605/Osf.Io/Pes2y).

## Statistical methods

All analyses were conducted using R software (R Core Team, 2021). We examined the effects of interest using logistic and linear mixed effects models from *lme4* (Bates et al., 2015). The models included fixed effects of headline type, reminder type, and correction classifications, where applicable, as well as by-participant and by-item random intercepts. We performed Wald’s χ^2^ hypothesis tests using the *Anova* function of the *car* package (Fox & Weisberg, 2019) and post-hoc comparisons controlling for multiple comparisons using Tukey’s HSD, implemented with the *emmeans* package (Lenth, 2021). The model specifications are available in the analysis scripts on the OSF (https://doi.org/10.17605/Osf.Io/Pes2y). The population estimates and 95% confidence intervals are derived from the models. The significance level was α = 0.05.

### Experiment 1: results

#### Phase 1: familiarity and perceived accuracy

Table 1 displays model-estimated familiarity and perceived accuracy ratings for headlines in phase 1. Familiarity ratings were relatively low on average and significantly higher for real than fake news, *t*(4899) = 4.07, *p* < 0.001. Perceived accuracy ratings were more intermediate on average and significantly higher for real than fake news, *t*(4996) = 12.60, *p* < 0.001. Together, these results show that participants could generally discern real from fake news despite only having some pre-existing knowledge of the headlines.

**Table 1:** Baseline familiarity and perceived accuracy ratings in phase 1

		Headline type	
Experiment	Measure	Real news	Fake news
Experiment 1	Familiarity	2.18 [2.06, 2.30]	2.08 [1.96, 2.20]
	Perceived accuracy	2.68 [2.60, 2.76]	2.41 [2.33, 2.49]
Experiment 2	Perceived accuracy	2.66 [2.60, 2.73]	2.38 [2.31, 2.44]
Experiment 3	Perceived accuracy	2.65 [2.59, 2.72]	2.41 [2.35, 2.48]

The values above are estimated marginal means from mixed effects models. 95% confidence intervals appear in brackets

#### Phase 2: reminder retrieval accuracy

**Fig. 3:**
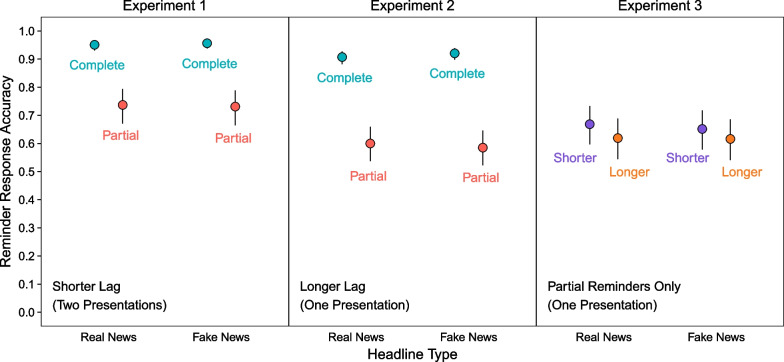
*Reminder Retrieval Accuracy in Phase 2*. The points are marginal means from mixed effects models. Error bars are 95% confidence intervals

We defined accurate reminder retrievals as the highest-confidence recognition (3, “Completely Remember”) for complete reminders and correct recall of phase 1 details for partial reminders. Figure 3 (left panel) shows greater retrieval accuracy for complete than partial reminders. A model with reminder and headline type as fixed effects indicated a significant effect of reminder type, χ^2^(1) = 443.03, *p* < 0.001, and no other significant effects, largest χ^2^(1) = 0.71, *p* = 0.40. These results show that reminders cued retrieval better when they involved recognition than recall.

#### Phase 3: recall and correction classifications

##### Real news recall

**Fig. 4:**
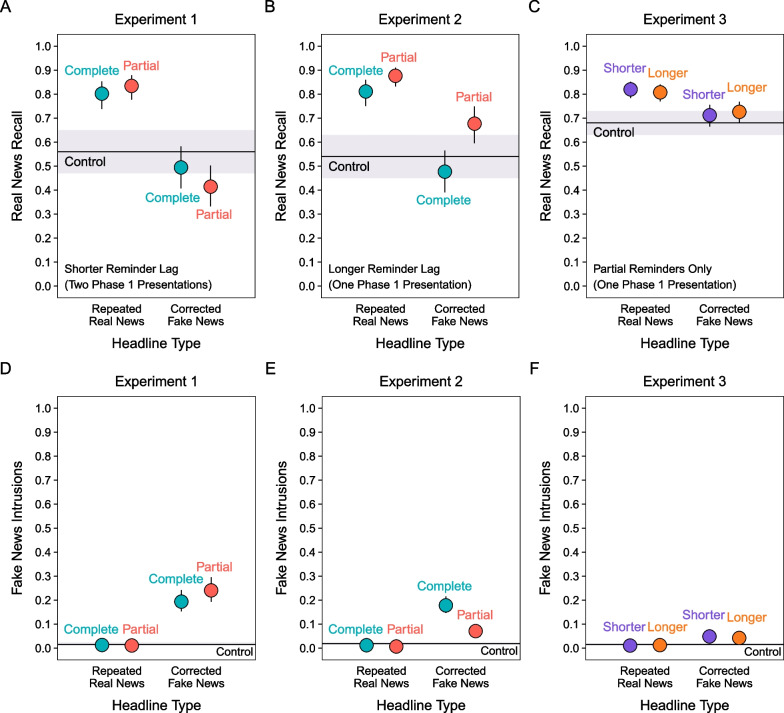
*Real News Recall and Intrusions of Fake News in Phase 3*. The points and horizontal lines are marginal means from mixed effects models. Error bars and shaded regions are 95% confidence intervals

Figure 4A shows real news recall across conditions. We assessed phase 1 exposure and reminder-type effects by comparing the four experimental conditions with the control condition. A model with a fixed effect of item type including all five conditions indicated a significant effect, χ^2^(4) = 532.07, *p* < 0.001. Repeating real news led to higher recall than in the control condition for both reminder types, smallest *z* ratio = 11.31, *p* < 0.001, with no difference between reminder types, *z* ratio = 2.02, *p* = 0.26. In contrast, complete fake news reminders led to lower recall than in the control condition for both reminder types, smallest *z* ratio = 2.93, *p* = 0.03, with recall being significantly lower for partial than complete reminders, *z* ratio = 3.50, *p* < 0.01. These results show that reminders counteracted proactive interference from fake news exposure better when they cued recognition rather than recall of fake news.

##### Intrusions of fake news

Figure 4D shows intrusions of fake news across all conditions. In these comparisons, the repeated real news and control conditions served as baseline indices of responding with fake news details that never appeared in the experiment. Those intrusions were therefore extra-experimental. In contrast, intrusions from phase 1 in the corrected fake news conditions were intra-experimental. A model with a fixed effect of item type including all five conditions indicated a significant effect, χ^2^(4) = 582.71, *p* < 0.001. Fake news details intruded in the correction conditions more following partial than complete reminders, *z* ratio = 2.81, *p* = 0.04. These intra-experimental intrusions far exceeded the extra-experimental intrusions in the other conditions, smallest *z* ratio = 14.20, *p* < 0.001. These results align with real news recall in showing less proactive interference following complete than partial reminders.

##### Correction classification and fake news recall

**Fig. 5:**
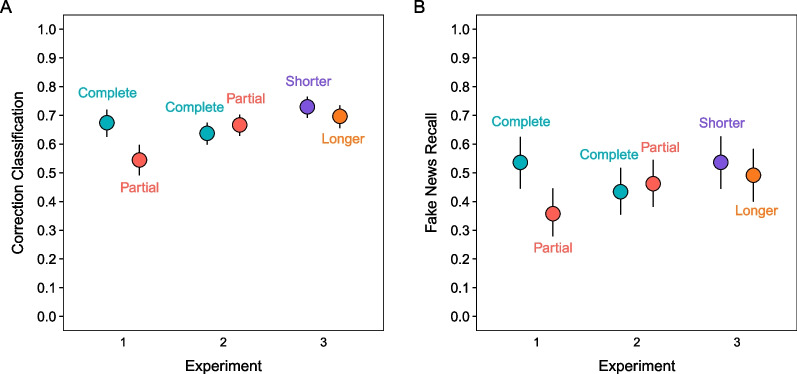
*Memory for Corrections and Fake News Recall for the Corrected Fake News Conditions*. The points are marginal means from mixed effects models. Error bars are 95% confidence intervals. Values for the repeated and control conditions are not displayed to emphasize differences in memory for corrections across experiments

Figure 5A shows correction classifications that participants gave in phase 3 to indicate their memory for fake news headlines being corrected in phase 2. The figure only displays the conditions with fake news that appeared in phase 1. A model with a fixed effect of the reminder–headline-type conditions indicated a significant effect, χ^2^(4) = 1546.40, *p* < 0.001. Participants classified corrections more accurately following complete than partial reminders, *z* ratio = 6.34, *p* < 0.001. These probabilities far exceeded incorrect classification probabilities in the other conditions (< 0.06), smallest *z* ratio = 23.06, *p* < 0.001. Figure 5B shows fake news recall only for the conditions with fake news in phase 1. We did not model recall in the other conditions because the probabilities of fake news recall were near zero (< 0.01). A model with a fixed effect of the reminder-type conditions indicated a significant effect, χ^2^(1) = 57.25, *p* < 0.001, showing that recall for fake details was higher following complete than partial reminders. Collectively, these results also correspond with the previous two outcomes showing better memory accuracy following complete than partial reminders.

#### Phase 3: recall for corrections of fake news conditioned on correction classifications

For corrections of fake news, we determined the extent to which reminder-induced retrieval promoted integrative encoding and familiarity backfire by characterizing retrieval dependencies among recall and correction classification measures. To do this, we first examined real news recall and intrusions of fake news for items with accurate reminder retrieval, conditioned on the three following types of correction classifications at test. *Fake News Recalled* refers to instances when participants remembered that fake news had been corrected in phase 2 and subsequently recalled the fake news details in phase 3. *Correction Remembered* refers to instances when participants remembered that fake news had been corrected in phase 2 but did not subsequently recall the fake news details in phase 3. *Correction Not Remembered* refers to instances when participants did not remember that fake news had been corrected in phase 2. We contextualize how these conditional probabilities contribute to overall responding by presenting cell counts (and proportions) in Table 2.

**Table 2:** Observation counts (and proportions) for corrections of fake news conditioned on reminders, lags, reminder retrieval, and correction classification

			Correction classification		
Experiment	Reminder/Lag	Reminder retrieval	Fake news recalled	Remembered not recalled	Not remembered
Experiment 1	Complete/Shorter	Correct	660 (.51)	135 (.10)	402 (.31)
		Incorrect	28 (.02)	26 (.02)	51 (.04)
	Partial/Shorter	Correct	460 (.35)	54 (.04)	377 (.29)
		Incorrect	71 (.06)	116 (.09)	224 (.17)
Experiment 2	Complete/Longer	Correct	1221 (.44)	353 (.12)	861 (.31)
		Incorrect	85 (.03)	73 (.03)	193 (.07)
	Partial/Longer	Correct	1143 (.41)	98 (.04)	336 (.12)
		Incorrect	200 (.07)	360 (.13))	649 (.23)
Experiment 3	Partial/Shorter	Correct	1341 (.47)	129 (.04)	329 (.11)
		Incorrect	218 (.08)	336 (.12)	531 (.18)
	Partial/Longer	Correct	1258 (.44)	116 (.04)	318 (.11)
		Incorrect	212 (.07)	358 (.12)	622 (.22)

##### Complete and partial reminders conditioned on correction classifications

**Fig. 6:**
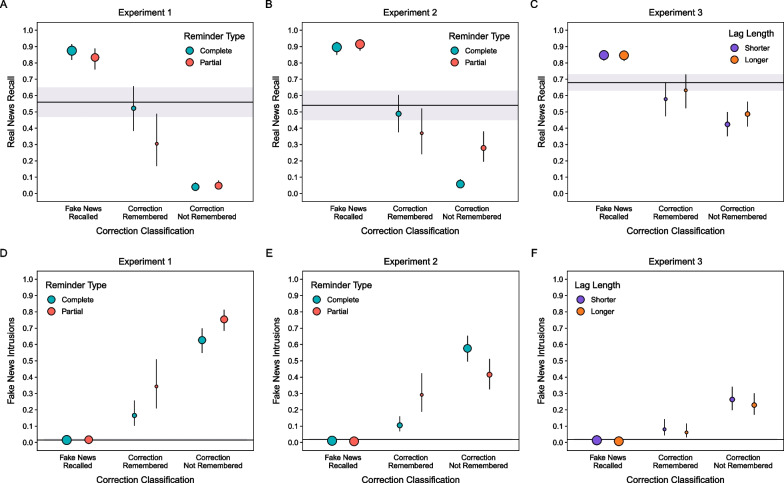
*Phase 3 Recall Following Accurate Reminder Retrieval Conditioned on Correction Classifications*. The points and horizontal lines are marginal means from mixed effects models. The point sizes indicate the relative differences in the number of observations in each cell. Error bars and shaded regions are 95% confidence intervals

Figure 6A shows real news recall for corrections of fake news for each reminder type conditioned on correction classifications. A model including these two variables as fixed effects indicated a significant effect of correction classification, χ^2^(2) = 515.58, *p* < 0.001, and no other significant effects, largest χ^2^(2) = 4.83, *p* = 0.09. For accurate classifications, real news recall was significantly higher when fake news was recalled than when it was not recalled, *z* ratio = 8.96, *p* < 0.001. When fake news was not recalled, real news recall was significantly higher when corrections were remembered than when they were not, *z* ratio = 9.92, *p* < 0.001. These results replicate the retrieval dependencies in studies of fake news correction effects on memory (Kemp et al., 2022a, b; Wahlheim et al., 2020) and indicate that both reminder types affected subsequent recall comparably when they cued retrieval of fake news details. Taken with the higher retrieval accuracy for complete and partial reminders above, these results indicate that overall differences in real news recall reflected the extent to which accurate reminder retrieval promoted associative encoding and later recollection.

Figure 6D shows intra-experimental intrusions of fake news for each reminder type conditioned on correction classifications. We used a model with these variables as fixed effects, excluding the fake news recall cell with intrusion rates near zero. A significant effect of reminder type, χ^2^(1) = 13.89, *p* < 0.001, showed more intrusions for partial than complete reminders; a significant effect of correction classification, χ^2^(1) = 74.69, *p* < 0.001, showed more intrusions when corrections were not remembered than when they were. There was no significant interaction, χ^2^(1) = 1.01, *p* = 0.31. These results showed that recall-based retrieval of fake news led to more proactive interference than recognition-based retrieval. Proactive interference was comparably reduced for both retrievals when corrections were remembered.

##### Partial reminders conditioned on reminder retrieval and correction classifications

We then examined real news recall and intrusions of fake news for only partial reminders conditioned on correction classifications and reminder retrieval accuracy. We could not conduct this analysis for the complete reminders because reminder recognition was near ceiling. This analysis allowed us to determine how recalling fake news before reading real news led to associative encoding but also familiarity-based errors during phase 3 recall. We do not elaborate on main effects of correction classifications redundant with those above.

**Fig. 7:**
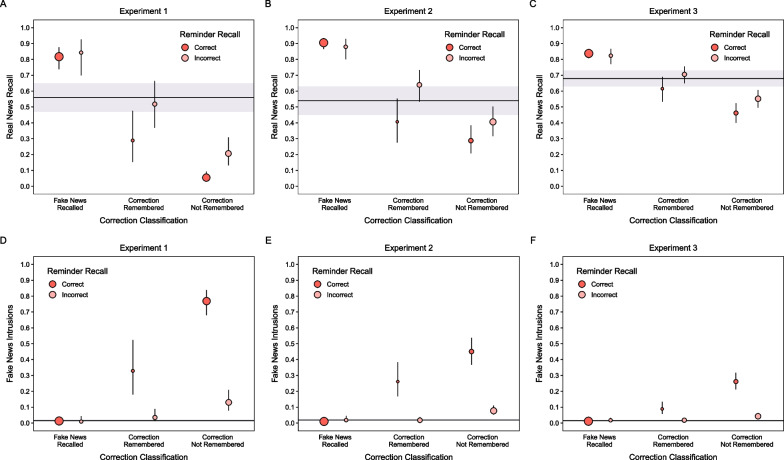
*Phase 3 Recall Following for Partial Reminders Conditioned on Reminder Retrieval and Correction Classifications*. The points and horizontal lines are marginal means from mixed effects models. The point sizes indicate the relative differences in the number of observations in each cell. Error bars and shaded regions are 95% confidence intervals

Figure 7A shows real news recall for corrections of fake news for partial reminders conditioned on reminder retrieval and correction classifications. A model including these two variables as fixed effects indicated significant effects of reminder retrieval, χ^2^(1) = 21.01, *p* < 0.001, and correction classification, χ^2^(2) = 252.42, *p* < 0.001, and a significant interaction, χ^2^(2) = 6.83, *p* = 0.03. When corrections were not remembered, real news recall was significantly higher when fake news had not been recalled during phase 2 reminders than when it had been, *z* ratio = 4.88, *p* < 0.001. There were no significant effects of reminder recall for the other classifications, largest *z* ratio = 2.20, *p* = 0.24. Retrieval practice of fake news led to more proactive interference when people could not remember that it was corrected.

Figure 7D shows intra-experimental intrusions of fake news for partial reminders conditioned on reminder retrieval and correction classifications. A model included these two variables as fixed effects without the fake news recalled cell for intrusions, which were near zero. A significant effect of reminder retrieval, χ^2^(1) = 113.62, *p* < 0.001, showed more intrusions when participants had recalled fake news in phase 2; a significant effect of correction classification, χ^2^(1) = 25.77, *p* < 0.001, showed more intrusions when participant did not remember fake news being corrected in phase 2. There was no significant interaction, χ^2^(1) = 0.72, *p* = 0.40. These findings suggest that retrieval practice of fake news led to familiarity-based intrusions when participants did not recollect that real news corrected fake news. Not surprising, participants inability to recall fake news during phase 2 reminders indicated that those details were highly inaccessible and therefore unlikely to later intrude.

### Experiment 1: discussion

Experiment 1 showed that fake news reminder effects varied with the details included. Complete reminder recognition prompts led to less overall proactive interference than partial reminder-cued recall prompts. This occurred even though fake news exposure was greater for complete than partial reminders. Complete reminders promoted recollection of fake news details and that they were corrected more than partial reminders. Such recollection was associated with enhanced subsequent memory for real news, especially when fake news was also recalled. These outcomes were generally more consistent with the integration than familiarity backfire view. However, reminder-cued retrieval also led to familiarity-based intrusions at test when participants did not use recollection of corrections to oppose those intrusions. This unwanted influence of familiarity was especially apparent when partial reminder recall led to more intrusions on the final test. The finding that reminder-cued retrieval enhances and impairs memory is consistent with work suggesting memory updating performance reflects a trade-off between recollection- and familiarity-based retrievals (for a review, see Wahlheim et al., 2021).

## Experiment 2

Experiment 1 examined complete and partial fake news reminder effects on memory for real and fake news headlines. Experiment 2 extended on those findings in two ways. We examined whether complete reminder benefits replicate when there is less fake news exposure and potential interference in phase 1. Experiment 1 showed that partial reminders led to overall proactive interference effects. Reducing fake news exposure may therefore minimize the negative consequences of retrieving fake news during reminders and later failing to recollect the fake detail and that it was corrected. We tested this hypothesis by (1) presenting phase 1 fake news headlines once and (2) adding a distractor task between phases 1 and 2. We expected that reducing phase 1 headline accessibility would eliminate the final recall difference between reminder conditions in Experiment 1. Our rationale was that partial reminders would be more sensitive to reductions in fake news accessibility because they do not include fake news details whereas complete reminders include those details.

We also examined fake news reminder effects on belief change. Conditions that improve memory sometimes improve belief accuracy (Wahlheim et al., 2020), but this is not always observed (Collier et al., 2023; Kemp et al., 2022a). Here, we compared perceived accuracy of recalled details in phase 3 with baseline ratings from phase 1. This indicates the extent to which initial beliefs contribute to subsequent memory accuracy for each type of reminder retrieval. We did not a priori hypothesize about belief change differences across reminder types. However, studies have shown improved belief accuracy when accuracy ratings are based on recollection that fake news was corrected (Kemp et al., 2022a; Wahlheim et al., 2020). We, therefore, expected greater differences in perceived accuracy between real news recall and fake news intrusions when participants remembered fake news being corrected.

### Experiment 2: method

#### Participants

Our stopping rule was to acquire usable data from at least 140 participants by testing all available participants in one semester. We based the sample size on a sensitivity analysis of the smallest effect size of interest from Experiment 1 (see Additional File 1, SI Section 3). We included usable data from 199 Duke University students (132 women, 58 men, 2 non-binary, and 7 unidentified) ages 18–22 (*M* = 19.10, *SD* = 1.10) who participated for course credit. We excluded data from one participant who only responded to 50% of the partial reminders.

#### Design, materials, and procedure

Experiment 2 used the same design and materials as Experiment 1, but we changed some procedural details. These changes are described in the following paragraph and illustrated in a procedural schematic provided in Fig. 2 (middle row).

We included five within-subjects conditions with four conditions emerging from a 2 × 2 crossed factorial design including the same headline and reminder types as before. As in Experiment 1, participants viewed real and fake news headlines. However, each headline appeared once, and participants rated their accuracy. Also diverging from Experiment 1, a distractor task between phases 1 and 2 required participants to answer 56 general knowledge questions that probed common misconceptions, adapted from Sinclair et al. (2020). The complete set of questions and response options are available on the OSF (https://doi.org/10.17605/Osf.Io/Pes2y). The questions appeared for 8 s each in random order with two response options below. Participants pressed “A” or “B” to choose an option and advanced trials using the mouse to click a button during the ISI. Phase 2 was the same as Experiment 1; participants saw real news headlines that corrected fake news or affirmed real news after complete or partial reminders. A control condition showed real news that did not appear in phase 1 and did not follow a reminder in phase 2. Phase 3 followed Experiment 1, but after attempting to recall a real news detail, participants rated the accuracy of the detail using the scale from phase 1.

### Experiment 2: results

#### Phase 1: perceived accuracy

Table 1 shows that perceived accuracy estimates for headlines in phase 1 were again intermediate and significantly higher for real than fake news, *t*(10,476) = 19.38, *p* < 0.001, indicating that participants could generally discern real from fake news.

#### Phase 2: reminder retrieval accuracy

Replicating Experiment 1, Fig. 3 (middle panel) shows more accurate retrieval for complete than partial reminders. A model with reminder and headline type as fixed effects indicated a significant effect of reminder, χ^2^(1) = 1233.55, *p* < 0.001, no significant effect of headline, χ^2^(1) = 0.12, *p* = 0.73, and a significant interaction, χ^2^(1) = 4.68, *p* = 0.03. These results show that as in Experiment 1, recognition accuracy was higher than recall accuracy. However, accuracy was significantly higher for complete reminders of fake than real news headlines, *z* ratio = 1.96, *p* < 0.05, and not significantly different between headline types for partial reminders, *z* ratio = 0.99, *p* = 0.33. We cannot explain this interaction.

#### Phase 3: recall and correction classifications

##### Real news recall

Figure 4B shows real news recall. A model with a fixed effect including all conditions indicated a significant effect, χ^2^(4) = 994.02, *p* < 0.001. As in Experiment 1, repeating real news led to higher recall than in the control condition for both reminders, smallest *z* ratio = 18.10, *p* < 0.001. Contrary to Experiment 1, recall was significantly higher for partial than complete reminders, *z* ratio = 6.37, *p* < 0.001. This effect is consistent with the documented larger benefits of recall- than recognition-based retrieval practice (Bjork & Whitten, 1974). Importantly, fake news reminders showed a pattern that was the opposite of the results observed in Experiment 1. Compared to the control condition, complete reminders led to lower recall, *z* ratio = 3.81, *p* = 0.001, whereas partial reminders led to higher recall, *z* ratio = 8.62, *p* < 0.001. Taken together, the findings from Experiments 1 and 2 show that real news recall depended on the interaction of fake news accessibility and reminder cuing efficacy.

##### Intrusions of fake news

Figure 4E shows intrusions of fake news. As a reminder, intrusions in the repeated real news and control conditions were extra-experimental; intrusions in the corrected fake news conditions were intra-experimental. A model with a fixed effect of all conditions indicated a significant effect, χ^2^(4) = 950.19, *p* < 0.001. Contrary to Experiment 1, for corrections, more fake news details intruded for complete than partial reminders, *z* ratio = 12.84, *p* < 0.001. Consistent with Experiment 1, intra-experimental intrusions exceeded extra-experimental intrusions, smallest *z* ratio = 11.16, *p* < 0.001. These results align with real news recall in showing more accurate real news recall after partial than complete reminders.

##### Correction classification and fake news recall

Figure 5A shows correction classifications in phase 3. A model with a fixed effect including all conditions indicated a significant effect, χ^2^(4) = 3516.10, *p* < 0.001. Unlike Experiment 1, correction classifications did not differ between reminder types, smallest *z* ratio = 2.14, *p* = 0.20. These accurate classifications exceeded inaccurate classifications in the other conditions (< 0.09), smallest *z* ratio = 37.07, *p* < 0.001. Unlike Experiment 1, the patterns of correction classification and real news recall did not fully align, suggesting that reminder-type effects on phase 2 encoding did not completely account for later recall differences. Figure 5B shows fake news recall for the correction conditions. As in Experiment 1, we did not model the repeated real news and control conditions with near-zero probabilities (< 0.01). A model with a fixed effect of reminder type indicated no effect, χ^2^(1) = 2.88, *p* = 0.09, paralleling the lack of difference in correction classifications. Taken together with Experiment 1, these results suggest that for partial reminders, the lag between initial fake news exposure and reminders improved recollection of fake news and that it was corrected. To verify the qualitative differences in these patterns, we fit separate exploratory models to predict correction classification and fake news recall, including *reminder type* and *experiment* as fixed effects. Both models showed a significant interaction, smallest χ^2^(4) = 48.74, *p* < 0.001, showing that for partial reminders, correction classification and fake news recall was significantly higher in Experiment 2 than Experiment 1, smallest *z* ratio = 2.38, *p* = 0.02. For complete reminders, correction classification did not differ between the two experiments, *z* ratio = 1.43, *p* = 0.15, but fake news recall was significantly higher in Experiment 1 than Experiment 2, *z* ratio = 2.14, *p* = 0.03.

#### Phase 3: recall for corrections of fake news conditioned on correction classifications

##### Complete and partial reminders conditioned on correction classifications

As in Experiment 1, the conditional recall analyses only included observations for which participants responded accurately to reminders. Table 2 shows the cell counts (and proportions). Figure 6B shows real news recall for corrections of fake news for each reminder type conditioned on correction classifications. A model including these two variables as fixed effects indicated significant effects of reminder, χ^2^(1) = 33.73, *p* < 0.001, and classification, χ^2^(2) = 792.58, *p* < 0.001, and a significant interaction, χ^2^(2) = 58.78, *p* < 0.001. As in Experiment 1, following accurate classifications, real news recall was significantly higher when fake news was recalled than when it was not, *z* ratio = 14.37, *p* < 0.001. When fake news was not recalled, real news recall was significantly higher when corrections were remembered than when they were not, *z* ratio = 8.50, *p* < 0.001. Contrary to Experiment 1, when corrections were not remembered, real news recall was higher for partial than complete reminders, *z* ratio = 9.37, *p* < 0.001; when corrections were remembered, regardless of whether fake news was recalled, real news recall did not differ between reminders, largest *z* ratio = 1.66, *p* = 0.56. These results replicate the retrieval dependencies shown before, but uniquely show a selective reminder-driven recall difference when changes were not remembered.

Figure 6E shows convergence in such reminder effects in conditional analyses of intrusions of fake news. We compared these effects using a model including reminder type and correction classification as fixed effects but excluding the cell with fake news recall and intrusion rates near zero. The model indicated no significant effect of reminder, χ^2^(1) = 3.57, *p* = 0.06, a significant effect of classification, χ^2^(1) = 98.10, *p* < 0.001, and a significant interaction, χ^2^(1) = 29.45, *p* < 0.001. Contrary to Experiment 1, practice retrieving fake news led to less proactive interference following partial than complete reminders when corrections of fake news were not recollected as such, *z* ratio = 4.17, *p* < 0.001. Taken together with the real news recall results, these results indicate that partial reminders created less familiarity-based interference, leading to better memory accuracy when recollection was less available.

##### Partial reminders conditioned on reminder retrieval and correction classifications

We then examined real news recall and intrusions of fake news for only partial reminders, conditioned on correction classifications and earlier reminder retrieval accuracy. As in Experiment 1, we could not conduct this analysis for the complete reminders because reminder recognition was near ceiling, despite the reduction in phase 1 headline accessibility. We do not elaborate on main effects of correction classifications redundant with those above.

Figure 7B shows real news recall following partial fake news reminders. This recall is conditioned on reminder retrieval and correction classifications. A model including these variables as fixed effects indicated significant effects of reminder retrieval, χ^2^(1) = 9.02, *p* < 0.01, and classification, χ^2^(2) = 343.40, *p* < 0.001, and a significant interaction, χ^2^(2) = 9.71, *p* = 0.01. Real news recall in phase 3 did not differ based on fake news reminder recall in phase 2 when fake news was recalled in phase 3, *z* ratio = 0.95, *p* = 0.93. However, fake news reminder recall in phase 2 led to improved real news recall in phase 3 when fake news was not recalled in phase 3, smallest *z* ratio = 3.26, *p* = 0.01.

Figure 7E shows intrusions of fake news for partial fake news reminders. These intrusions are conditioned on reminder retrieval and correction classifications. As in the conditional intrusion analyses, we compared these probabilities using a model excluding fake news recall in phase 3 with intrusions that were near zero. A model including these two variables as fixed effects indicated significant effects of reminder retrieval, χ^2^(1) = 144.93, *p* < 0.001, and classification, χ^2^(1) = 19.78, *p* < 0.001, and no significant interaction, χ^2^(1) = 2.17, *p* = 0.14. As in Experiment 1, these results showed that intrusion production depended almost entirely on fake news being recalled for partial reminders in phase 2. Taken with conditional real news recall, these results show that practice retrieving fake news led to familiarity-based interference errors that were nearly absent when fake new reminders did not cue recall.

#### Phase 1 and 3: headline beliefs conditioned on subsequent memory accuracy

**Fig. 8:**
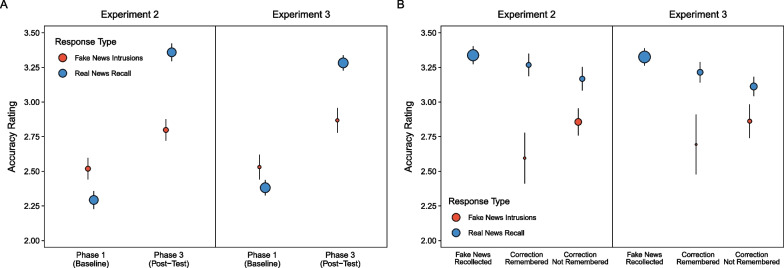
*Accuracy Ratings Indicating Belief Change from Phase 1 to Phase 3*. The points are marginal means from mixed effects models. Error bars are 95% confidence intervals

Research on news headline corrections showed relationships between memory and perceived accuracy (Kemp et al., 2022a, 2022b; Wahlheim et al., 2020). We extended on that work by examining the extent to which differences in recall of fake news corrections were accompanied by changes in accuracy ratings for entire headlines phase 1 and recalled details in phase 3. Figure 8A (left panel) shows accuracy ratings in phases 1 and 3 conditioned on the type of response output in phase 3. Table 3 displays the results from a model including phase, response type, and reminder type as fixed effects. Of primary interest, there was a significant phase-by-response interaction. In phase 1, the perceived accuracy of fake news was significantly higher for headlines that led to intrusions than real news recall, *z* ratio = 7.53, *p* < 0.001; but in phase 3, perceived accuracy was significantly higher for real news recall than intrusions of fake news, *z* ratio = 18.70, *p* < 0.001. These results suggest that, on average, participants could discern real from fake details that they recalled. These results also suggest that initial perceptions of accuracy affected subsequent recall reports because intrusions originated from headlines that were originally perceived as more accurate. There was also a significant, but ambiguous, phase-by-reminder interaction; pairwise comparisons revealed no significant differences in perceived accuracy between reminder types within phases 1 and 3, largest *z* ratio = 1.80, *p* = 0.27.

**Table 3:** Model results for accuracy comparisons in phases 1 and 3: experiments 2 and 3

Experiment	Effect	χ^2^	*df*	*p*
Experiment 2	Phase	3060.92	1	< .001
	Response	55.10	1	< .001
	Reminder	0.39	1	= .53
	Phase × Response	413.68	1	< .001
	Phase × Reminder	8.79	1	= .003
	Response × Reminder	1.41	1	= .24
	Phase × Response × Reminder	0.01	1	= .97
Experiment 3	Phase	3108.30	1	< .001
	Response	19.69	1	< .001
	Lag	0.10	1	= .75
	Phase × Response	111.41	1	< .001
	Phase × Lag	0.04	1	= .83
	Response × Lag	1.72	1	= .19
	Phase × Response × Lag	0.55	1	= .46

Figure 8B (left panel) shows accuracy ratings in phase 3 conditioned on correction classifications. We excluded intrusions when fake news was also recalled because such responses were redundant and rare. We collapsed over reminder type because it did not interact with other variables. We used separate models for each response type. Both models indicated a significant classification effect, smallest χ^2^(1) = 8.32, *p* = 0.001, and no other significant effects, largest χ^2^(1) = 0.61, *p* = 0.44. For real news recall, there was no significant difference in perceived accuracy for the classifications with remembered corrections, *z* ratio = 2.26, *p* = 0.06, but perceived accuracy was significantly lower for corrections that were not remembered than the other classifications, smallest *z* ratio = 2.42, *p* = 0.04. For intrusions of fake news, perceived accuracy was significantly lower for remembered than not remembered corrections, *z* ratio = 2.88, *p* < 0.01. Together, these results show that participants discerned real from fake details, especially when they could remember corrections having occurred.

### Experiment 2: discussion

Replicating Experiment 1, retrieving fake news during reminders and recollecting corrections at test were associated with better recall of real news and fewer intrusions of fake news. Contrary to Experiment 1, however, we found that proactive interference was greater after complete than partial reminders. In fact, partial reminders led to proactive facilitation, enhancing recall of real news. Fake news recall did not differ between reminder types and was associated with comparable proactive facilitation in both conditions. Contrary to Experiment 1, partial reminders seemed to promote more durable integration that supported recollection, relative to complete reminders. Additionally, failing to recollect corrections was associated with more fake news interference for complete than partial reminders. Two key differences in Experiment 2 may explain this reversal—fewer phase 1 fake news exposures and the delay between phases 1 and 2. The strength of initial encoding and/or accessibility of fake news may moderate reminder effects.

The accuracy ratings revealed two key outcomes. First, fake news in phase 1 seemed less accurate when associated with later real news recall than intrusions. However, participants still discerned retrieved real from fake news. These findings suggest the intrinsic believability of fake news undermined the efficacy of corrections. Second, participants could discern real from fake news better when they remembered corrections. This implicated a role for conscious memory for experimenter-provided veracity information in belief updating.

## Experiment 3

The results from Experiments 1 and 2 showed that varying the retrieval demands of reminders led to differences in memory updating. In both experiments, complete reminders led to proactive interference effects, impairing memory for real news details. In contrast, the effects of partial reminders differed across experiments: Partial reminders caused greater proactive interference in Experiment 1, but produced substantial proactive facilitation in Experiment 2. These opposite effects may be explained by two methodological differences between Experiment 1 and Experiment 2. In Experiment 2, we reduced the accessibility of memories of fake news by 1) reducing the number of initial exposures during the encoding phase and 2) increasing the delay between the encoding phase and the reminder/correction phase. Experiment 3 investigated the role of the latter by varying the delay period between initial exposure (phase 1) and correction (phase 2).

In Experiment 3, participants were exposed to fake news headlines only once during phase 1 (as in Experiment 2), either before or after a trivia question task that served as a filler task. As a result, items encountered before the filler task had a longer delay between initial exposure and correction, whereas items that appeared after the filler task had a shorter delay between initial exposure and correction. This procedure allowed us to test competing hypotheses regarding the effects of lags between fake news exposure and reminders on proactive memory effects. The familiarity backfire view predicts that a longer lag between initial fake news exposure and partial reminders will reduce reminder-cued retrieval and proactive interference. In contrast, an integrative encoding view predicts that a shorter lag will lead to higher reminder-cued retrieval, because increasing memory accessibility would promote recollection and thus facilitate integrative encoding and accurate recall of news details. We did not develop a priori hypotheses about the effects of lag length on perceived accuracy.

### Experiment 3: method

#### Participants

Our stopping rule was to test all available participants in one semester. Given the substantive design change in Experiment 3 relative to its predecessors, we did not a priori identify an effect size of interest. The number of participants available at the beginning of the semester indicated that we could expect usable data from approximately 130 participants. We considered this an acceptable minimum sample size because it provides adequate power to detect a small-medium effect. We included usable data from 206 Duke University students (136 women, 58 men, 3 other, and 9 unidentified) ages 18 – 35 (*M* = 19.50, *SD* = 1.65) who participated for course credit. Nine participants did not provide their age. We excluded data from one participant who only responded to 60% of the partial reminders.

#### Design, materials, and procedure

Experiment 3 used the materials from Experiment 1 but only included partial reminders. We included five within-subjects conditions with four emerging from a 2 × 2 crossed factorial design and manipulated headline types as before. We crossed headline types with lag length by varying the number of events between phases 1 and 2. We divided phase 1 into two parts: In phase 1a, half of the headlines appeared before a distractor task (longer lag condition), while in phase 1b, the other half appeared after a distractor task (shorter lag condition). The distractor task that we inserted between phases 1a and 1b was the general knowledge task from Experiment 2. Figure 2 (bottom panel, Experiment 3) displays a procedural schematic.

### Experiment 3: results

#### Phase 1: perceived accuracy

Table 1 shows that perceived accuracy estimates for headlines in phase 1 were again intermediate and significantly higher for real than fake news, *t*(10,733) = 16.45, *p* < 0.001, indicating that participants could generally discern real from fake news.

#### Phase 2: reminder retrieval accuracy

Figure 3 (right panel) shows greater reminder retrieval accuracy for those appearing after shorter than longer lags. A model with lag length and headline type as fixed effects indicated a significant effect of lag length, χ^2^(1) = 16.49, *p* < 0.001, and no other significant effects, largest χ^2^(1) = 0.95, *p* = 0.33, showing that reminders cued retrieval more effectively when fewer events intervened between the original headlines and reminder cues.

#### Phase 3: recall and correction classifications

##### Real news recall

Figure 4C shows real news recall. A model with a fixed effect including all conditions indicated a significant effect, χ^2^(4) = 204.20, *p* < 0.001. Repeating real news led to higher recall than in the control condition for both lags, smallest *z* ratio = 10.31, *p* < 0.001, with recall not differing between lags, *z* ratio = 1.20, *p* = 0.75. Fake news reminders led to higher real news recall than in the control condition for the longer lag, *z* ratio = 3.46, *p* < 0.01, but not shorter lag, *z* ratio = 2.36, *p* = 0.13, condition; however, recall did not differ between those lag conditions, *z* ratio = 1.10, *p* = 0.81. These results suggest that fake news reminder effects on real news recall depended on how well partial reminders cued retrievals.

##### Intrusions of fake news

Figure 4F shows intrusions of fake news. A model with a fixed effect including all conditions indicated a significant effect, χ^2^(4) = 207.03, *p* < 0.001. Intra-experimental intrusions were not significantly different between lags, *z* ratio = 1.35, *p* = 0.66, but were significantly higher than extra-experimental intrusions, smallest *z* ratio = 7.48, *p* < 0.001. These results parallel Experiment 2 in showing that with one initial fake news exposure in phase 1, intrusion rates were low when partial reminders preceded corrections.

##### Correction classification and fake news recall

Figure 5A shows correction classifications in phase 3. A model with a fixed effect including all conditions indicated a significant effect, χ^2^(4) = 4035.70, *p* < 0.001. Accurate classifications did not differ between lags, *z* ratio = 2.58, *p* = 0.07, and exceeded inaccurate classifications (< 0.08), smallest *z* ratio = 39.99, *p* < 0.001. Unlike Experiment 1, this pattern did not parallel real news recall. But like Experiment 2, these findings suggest that reminder effects on phase 2 encoding did not completely account for subsequent recall differences. Figure 5B shows fake news recall for the correction conditions. We did not model the other conditions due to near-zero probabilities (< 0.01). A model with a fixed effect of lag indicated significantly higher fake news recall for shorter than longer lags, χ^2^(1) = 6.88, *p* = 0.008, paralleling correction classifications and contrasting with real news recall. These results showed that higher reminder retrieval accuracy promoted subsequent memory for fake news.

#### Phase 3: recall for corrections of fake news conditioned on correction classifications

##### Partial reminders conditioned on correction classifications

As before, we restricted the conditional analyses to accurate reminder retrievals. Table 2 shows the cell counts (and proportions). Figure 6C shows real news recall for corrections of fake news. A model with lag and classification as fixed effects indicated no lag effect, χ^2^(1) = 0.84, *p* = 0.36, a significant classification effect, χ^2^(2) = 305.09, *p* < 0.001, and no interaction, χ^2^(2) = 1.92, *p* = 0.38. As before, when corrections were classified as such, real news recall was significantly higher when fake news was recalled than when it was not, *z* ratio = 8.02, *p* < 0.001. When fake news was not recalled, real news recall was significantly higher when corrections were remembered than when they were not, *z* ratio = 3.49, *p* = 0.001. The absence of lag effects here suggests that the opposing effects of partial reminders in Experiments 1 and 2 cannot be explained by the lag between phases 1 and 2.

Figure 6F shows convergence for intrusions of fake news. We used a model with lag and classification as fixed effects, excluding cells with fake news recall and rates near zero. The model indicated no lag effect, χ^2^(1) = 0.95, *p* = 0.33, a significant classification effect, χ^2^(1) = 34.62, *p* < 0.001, and no significant interaction, χ^2^(1) = 0.025, *p* = 0.88, showing that remembering corrections counteracted interference comparably at both lags.

##### Partial reminders conditioned on reminder retrieval and correction classifications

We then examined real news recall and intrusions of fake news conditioned on correction classifications and reminder retrieval accuracy. We do not elaborate on main effects of correction classifications redundant with those above.

Figure 7C shows real news recall for corrections of fake news conditioned on reminder retrieval and correction classifications. We used a model with those two fixed effects collapsed across lag because a first model that also included lag as a fixed effect showed no interactions with that variable, largest χ^2^(2) = 2.44, *p* = 0.30. The final model indicated significant effects of reminder retrieval, χ^2^(1) = 8.47, *p* = 0.004, and classification, χ^2^(2) = 342.75, *p* < 0.001, and a significant interaction, χ^2^(2) = 7.14, *p* = 0.03. When corrections were not remembered in phase 3, real news recall was higher when fake news was not recalled than when it was in phase 2, *z* ratio = 3.11, *p* = 0.02. Real news recall did not differ based on phase 2 fake news recall for the other classifications, largest *z* ratio = 2.42, *p* = 0.15.

Figure 7F shows intrusions of fake news conditioned on reminder retrieval and correction classifications. As before, we used a model excluding the cells with fake news recall because rates were near zero. A model with these two variables as fixed effects indicated significant effects of reminder retrieval, χ^2^(1) = 153.45, *p* < 0.001, and classification, χ^2^(1) = 37.15, *p* < 0.001, and no significant interaction, χ^2^(1) = 1.54, *p* = 0.22. As before, these results suggest that retrieving fake news during reminders led to proactive interference when recollection later failed. However, little, if any, proactive interference was present when fake news details were not retrieved for reminders.

#### Phase 1 and 3: headline beliefs conditioned on subsequent memory accuracy

We again examined how reminder-based corrections affected belief change from phase 1 to 3. Figure 8A (right panel) shows perceived accuracy ratings in phases 1 and 3 conditioned on whether participants recalled real news or intruded fake news details in phase 3. Table 3 displays the results from a model including phase, response type, and lag length as fixed effects. As in Experiment 2, there was a significant phase-by-response interaction. In phase 1, the perceived accuracy of fake news was significantly higher for headlines that led to intrusions than real news recall, *z* ratio = 3.74, *p* = 0.001; but in phase 3, perceived accuracy was significantly higher for real news recall than intrusions of fake news, *z* ratio = 10.41, *p* < 0.001. These results again suggest that participants were able to differentiate between recalled real and false details, and that intrusions more frequently originated from fake news that was more believable.

Figure 8B (right panel) shows accuracy ratings in phase 3 conditioned on correction classifications collapsed over lag length because that variable did not enter into a significant interaction. The model for real news recall indicated a significant classification effect, χ^2^(2) = 80.19, *p* < 0.001, and no other significant effects, largest χ^2^(2) = 2.95, *p* = 0.23. Perceived accuracy decreased significantly across recollected fake news, remembered corrections, and not remembered corrections, smallest *z* ratio = 3.34, *p* = 0.002. The model for intrusions of fake news indicated no significant effects, largest χ^2^(1) = 2.59, *p* = 0.11. Taken with the prior analyses of accuracy ratings, these results again suggest that participants were better able to discern real news recall from intrusions of fake news when they were able to recollect corrections. Note that despite the discrepancy in the statistical significance of effects across Experiments 2 and 3, the qualitative patterns were parallel.

### Experiment 3: discussion

Experiment 3 investigated the extent to which the duration of the lag between fake news exposure and correction could explain the opposing effects of partial reminders in Experiment 1 and Experiment 2. Two results suggested that lag played a minor role, but could not fully explain the differences between Experiments 1 and 2. First, partial reminders in Experiment 3 did not lead to proactive interference effects for real news recall, consistent with results for partial reminders in Experiment 2. This contrasted with Experiment 1, which showed that partial reminders induced proactive interference. Second, the longer lag increased real news recall and decreased fake news recall. This suggested that longer lags reduced contextual overlap and fake news accessibility. Longer lags may have reduced interference between fake news and real news. 

Overall, results from Experiment 3 suggest that the opposing effects observed in Experiments 1 and 2 may be more fully explained by the number of initial exposures to fake news during initial exposure, rather than by the duration of the lag between initial exposure and correction. However, this conclusion remains to be empirically verified. Taken with the prior experiments, these findings show that memory updating and perceived accuracy depended on the accessibility of fake news in memory and the type of reminder used during correction. Together, these factors determined whether real and fake details were associated and later recollected.

## General discussion

The present study examined how initial encoding of fake news and the type of reminder presented during correction influence memory and belief updating. We compared the effectiveness of two types of reminders presented before misinformation correction: complete reminders that reinstated false details (probing recognition) or partial reminders that omitted false details (probing recall). We found that either complete reminders or partial reminders can be more effective, depending on the accessibility of memories for fake news.

In Experiment 1, compared to partial reminders, complete reminders reduced proactive interference—enhancing recall of real news and reducing intrusions of fake news. In Experiment 2, this pattern was reversed—partial reminders led to proactive facilitation, enhancing misinformation correction. We hypothesized that this reversal could be explained by *memory accessibility*; in Experiment 2, participants had fewer initial exposures to fake news and experienced a longer lag between exposure and correction. In Experiment 3, we found that this reversal could not be explained by the lag between initial exposure and reminders. We conclude that the differing results observed in Experiments 1 and 2 were most likely due to differences in initial fake news exposure. Overall, we found that partial reminders were more effective at correcting fake news after a single exposure, but complete reminders were more effective when participants had repeatedly been exposed to fake news. Finally, all experiments also showed better memory and perceived accuracy when corrections were recollected, indicating a key role for associative encoding in subsequent memory for details and their veracity.

## Encoding/retrieval interactions and subsequent memory for fake news

The present results are relevant to the debate on how misinformation reminders affect correction efficacy. The *familiarity backfire view* posits that re-exposure to misinformation increases its familiarity, which may enhance its believability when people misattribute familiarity to truth (Pluviano et al., 2017, 2019; Schwarz et al., 2007; Skurnik et al., 2007). For example, Skurnik et al. (2007) found that participants who were exposed to myth corrections endorsed stronger beliefs in the myths on a later test, relative to participants who were not exposed. The idea that enhanced familiarity increases belief corresponds with the *illusory truth effect* showing that people infer greater truth after repeated exposure (Begg et al., 1992; Dechêne et al., 2010; Hasher et al., 1977). Together, these and other related findings suggest that avoiding misinformation repetition can reduce familiarity-based errors.

Contrary to this account, integration-based accounts propose that re-exposure to misinformation with corrections increases conflict detection and the co-activation necessary to encode the association of true and false details (Ecker et al., 2017), which can later be recollected (Kemp et al., 2022a, b; Wahlheim et al., 2020). Studies supporting this view have shown that providing misinformation reminders with retractions facilitates correct inferences (Ecker et al., 2017) as well as memory and belief updating (Wahlheim et al., 2020). This view meshes with research showing that detecting conflicts in texts promotes knowledge revision and mental model updating (Kendeou et al., 2014, 2019; Stadtler et al., 2013).

Our findings synthesize and clarify discrepant findings from prior studies by showing that the memory consequences of fake news reminders depend on the accessibility of fake news, how reminders elicit fake news retrieval, and the resulting effects on associative encoding and later recollection of corrections. Here, both reminder types improved real news recall when they led to accurate retrieval of fake news, as predicted by integrative encoding accounts (for a review, see Ecker et al., 2022). However, overall proactive memory effects of reminders depended on the rates of reminder success and later recollection. Conditional analyses suggested that associative encoding promoted memory updating for trials where corrections were recollected. But, when corrections were not recollected, familiarity-based intrusion errors occurred to the extent that reminders earlier promoted accurate fake new retrieval, consistent with the familiarity backfire view. This mixture of effects is consistent with prior studies on proactive effects of memory (for a review, see Wahlheim et al., 2021) and stresses the need for theories and application to consider a dual-process view positing that both integration leading to recollection and familiarity leading to errors can be present.

The present study also extended on the consequences of misinformation exposure from inferences and beliefs (Ecker et al., 2017; for a review see Lewandowsky et al., 2012), to episodic memory outcomes (Kemp et al., 2022a, b; Wahlheim et al., 2020). We found that the accessibility of fake news in memory influenced the effect of reminders during correction on memory updating. Recall of real news following corrections varied with reminder retrieval cues (recognition vs. recall) and the accessibility of fake news, especially prior fake news exposures (cf. Thomas et al., 2017). These findings contrast with the outcomes from traditional continued influence effect studies showing no differences in memory recall for misinformation and corrections under conditions that promote misinformation accessibility (Connor Desai & Reimers, 2023; Ecker et al., 2017; Johnson & Seifert, 1994; Pluviano et al., 2020; for a review, see Lewandowsky et al., 2012).

The discrepancies between these findings can partially be attributed to experimental design. Continued influence effect experiments often use a narrative paradigm, where participants read a story with details that are corrected after a few sentences. In contrast, the current study used a fake news correction paradigm (Wahlheim et al., 2020), where participants read individual statements that may later be updated. Narrative details may be easier to remember than disconnected statements because stories contain temporally ordered causal events, resembling everyday experiences (Graesser et al., 1991). Furthermore, in the narrative paradigm (Johnson & Seifert, 1994; Wilkes & Leatherbarrow, 1988), misinformation and its correction are often in the same paragraph, whereas the fake news correction paradigm presents fake news and corrections in separate phases, like list-learning paradigms in the verbal learning literature (for a review, see Wahlheim et al., 2021). Conflict detection and integrative encoding may be easier in narratives given the close temporal proximity and coherence, explaining the discrepant findings. However, fake news correction paradigms may be better suited for measuring proactive memory effects, as these paradigms provide ample opportunities to measure detection of and memory for corrections in association with recall of statements.

The present studies also relate to work on the effects of encoding and retrieval strength on misinformation correction. Prior studies have manipulated the strength of initial encoding or retractions by varying their presentation frequency (Ecker et al., 2011). This prior work showed that when misinformation was strongly encoded, stronger retractions more effectively reduce the continued influence effect, relative to weaker retractions. However, when misinformation was weakly encoded, strong retractions failed to eliminate the continued influence effect. Another study showed that reminders reiterating misinformation better mitigated the continued influence effect than subtle reminders (Ecker et al., 2017). These findings parallel our results. Complete reminders better corrected misinformation that was highly accessible after two initial exposures and a shorter lag before correction. In contrast, partial reminders better corrected less accessible misinformation after one initial exposure and a longer lag before correction. Together with the conditional results described above, these encoding/retrieval interactions highlight the idea that more accessible misinformation can improve or impair memory. Such proactive effects depend on how misinformation retrieval during reminders promotes associative encoding and later recollection to oppose familiarity.

The literature on the cognitive neuroscience of memory has also examined the effects of encoding strength and retrieval efficacy on memory updating. The *non-monotonic plasticity hypothesis* proposes that existing memories are transformed depending on their strength and how they are reactivated (Newman & Norman, 2010; Ritvo et al., 2019). Accordingly, moderately strong reactivation weakens the memory, promoting updating and differentiation. Strong reactivation strengthens the memory and promotes integrative encoding with new information. Other studies of memory reconsolidation have shown that partial reminders drive memory updating, perhaps by eliciting prediction error and promoting active recall (Sinclair & Barense, 2019). In contrast to these accounts, the present study suggested that complete and partial reminders both supported misinformation correction via integration to the extent that participants retrieved fake news details before correction. Reconciling these accounts will require further experiments that vary both initial fake news exposure frequency and reminder types (cf. Wahlheim et al., 2019), possibly in combination with neuroimaging.

## Perceived accuracy of real and fake news

We also examined associations between memory and beliefs in retrieved headline details. Although extensive work has examined the effects of misinformation exposure and corrections on belief updating (for a review, see Ecker et al., 2022), only a few studies have examined the role of memory in belief updating (Collier et al., 2023; Kemp, et al., 2022a; Swire-Thompson et al., 2023; Wahlheim et al., 2020). Typical belief updating paradigms collect belief ratings for misinformation statements, correct the misinformation, and then collect belief ratings again for the earlier statements (e.g., Swire et al., 2017; Swire-Thompson et al., 2023). Our approach differed by measuring perceived accuracy during initial fake news exposure and when participants tried to recall real news details after a correction phase. By measuring perceived accuracy of recalled details, we simulated the everyday experience of a person recalling and needing to evaluate the veracity of news details without rereading the original headline.

We found that participants discerned recalled real and fake details quite well, especially when they remembered that topics were corrected. These associations between memory for corrections and beliefs are compatible with other studies offering memory explanations for belief change (Kemp et al., 2022a; Swire-Thompson et al., 2023; Wahlheim et al., 2020) and accounts attributing the continued influence effect to selective retrieval of misinformation (for reviews, see Ecker et al., 2022; Sanderson & Ecker, 2020). One version of this account invokes a dual-process perspective (Jacoby, 1991, 1999) by assuming that reliance on misinformation persists when its familiarity is unopposed by recollection-based retrieval (Butterfuss & Kendeou, 2020; Ecker et al., 2011). Our findings add to this literature by suggesting that perceived accuracy depends on the extent to which fake news reminders enable successful integration and subsequent recollection of false and corrected information (Wahlheim et al., 2021; Kemp, et al., 2022a, 2022b).

We also found that fake news details that intruded during recall were initially more believable than correctly recalled real news details. This suggests that more believable fake news was harder to update, consistent with studies showing reduced correction efficacy for strongly believed misinformation (for a review, see Lewandowsky et al., 2012). However, the subjective confidence in the accuracy of reported details may have varied between correct real news and intrusions of fake news. Participants may have been less confident in the accuracy of intrusions but lowered their report criterion to respond even if they were guessing. This possibility could be tested with retrospective confidence judgments of perceived accuracy and the option to withhold responses. These task features allow for the strategic regulation of memory accuracy via monitoring and control processes (for a review, see Goldsmith & Koriat, 2007).

## Limitations and future directions

The present study had several limitations. The opposing results in Experiments 1 and 2 showing proactive interference reduction by complete reminders (Experiment 1) and then partial reminders (Experiment 2) led us to conclude that initial fake news accessibility determines which reminder type promotes more effective memory updating. The opposing pattern of results in Experiments 1 and 2 could be explained by either the number of fake news exposures during encoding or the lag between exposure and reminders. In Experiment 3, we examined the role of lag and found partial reminder effects like those observed in Experiment 2. In other words, lag cannot explain the discrepancy in results between Experiments 1 and 2. Experiment 3 thus provided indirect evidence that differences in initial fake news exposure (multiple exposures vs. single exposure) explained the discrepancy between Experiments 1 and 2. In future work, direct tests of this account will require simultaneous manipulations of fake news repetitions and reminder types.

In Experiment 1, both partial and complete reminders of fake news before corrections (real news) resulted in proactive interference effects in memory for real news, compared to a control condition where real news headlines appeared without corresponding fake news in phase 1 or a reminder in phase 2. The findings of interference effects could be misconstrued as suggesting that corrections with fake news reminders are detrimental. However, this would only be true if memory accuracy in these conditions was impaired compared to a contrast condition that included fake news in phase 1 and real news that appeared without reminders in phase 2. We did not include such a contrast condition in the present experiments because our focus was on direct comparisons of complete and partial reminders. It is also noteworthy that real news recall and belief accuracy was enhanced when successful fake news reminding promoted subsequent recollection that such news had been corrected. The present findings therefore illuminate the conditions under which one may expect fake news reminders to promote the sort of integrative encoding and subsequent memory necessary to promote updated memories. Future research could include no-reminder contrast conditions to gain a fuller understanding of the net effect of reminders on subsequent memory and beliefs.

Additionally, although we used everyday examples of fake news and corrections, in real-world settings people are exposed to news headlines differently than in our paradigm. For example, a small proportion of social media users are exposed to fake news very frequently, whereas most users rarely encounter fake news (Grinberg et al., 2019). While some unpublished manuscripts have begun to manipulate the proportion of misinformation to examine its influence (Altay et al., 2023; Butler et al., 2023; Orchinik et al., 2023), our task presented all participants with an equal distribution of real and fake news with instructions to consider the veracity of headlines and encode corrections. Warning people about the presence of misinformation can increase attention and scrutiny, leading to reduced perceptions of accuracy (Clayton et al., 2020; also see, Jalbert et al., 2020). Our findings may therefore underestimate the absolute perceived accuracy of fake news headlines in daily life, which may also have consequences for correction efficacy.

Lastly, it is important to note that memory for fake news in a lab paradigm may not generalize to real-world exposure to fake news. In the present study, we to enhance the ecological validity of our study by using genuine exemplars of fake news and a presentation format that mimics common fact-checks on social media. However, everyday memory for fake news and corrections is likely to be influenced by additional factors such as exposure, depth of encoding, attention, interest, ideology, knowledge, and sources. These factors have all been shown to influence memory in laboratory settings and may all contribute to memory for fake news in daily life. We could not account for all factors here, but future studies could do so. Our findings suggest that misinformation accessibility, whatever the cause, determines the efficacy of reminder cues for promoting memory updating. In the wild, it is challenging to track the factors that determine such accessibility to accurately recommend correction techniques.

## Conclusion

The present study examined how initial fake news exposure and reminder type before corrections affected memory and belief updating. Complete reminders better supported updating after repeated exposure to fake news, whereas partial reminders better supported updating after a single exposure. Our findings suggest that reminders can promote integrative encoding and memory updating, but the costs and benefits of reminders depend on both encoding and retrieval factors. The strength or accessibility of a memory interacts with the type of reminder to influence associative encoding of real and fake news as well as later recollection. These findings have practical implications for when to use particular reminders. After high fake news exposure, such as repeatedly encountering viral misinformation on social media, repeating the entire false claim during correction may support memory and belief updating. However, after light exposure to fake news, such as glancing at a headline, repeating only part of the false claim and encouraging self-generated retrieval may better support correction. Finally, the interactions between memory and beliefs observed in the present studies highlighted how enhancing memory for corrections can also sometimes improve belief accuracy.

### Supplementary Information

**Additional file 1**: Supplementary Section 1–3 and Figures S1–S3.

## Data Availability

The stimuli, data, and analysis scripts have been made publicly available via OSF (https://doi.org/10.17605/Osf.Io/Pes2y).
