# Supplementary material for: Memory and belief updating following complete and partial reminders of fake news
Source: Cogn Res Princ Implic. 2024 May 7;9:28. doi: 10.1186/s41235-024-00546-w (PMC11076432; doi:10.1186/s41235-024-00546-w)
Supplement: Supplementary file 1 — Additional file 1: Supplementary Section 1–3 and Figures S1–S3. [file 41235_2024_546_MOESM1_ESM.docx]

**Supplementary Information**

**1. Power Analysis for Experiment 1**

When planning Experiment 1, we used a standardized effect size estimate from the smallest effect of interest in (Wahlheim et al., 2020; Experiment 1) as a basis for a power analysis. The smallest effect size of interest in that study corresponded to the difference in correct real news recall in Phase 3 between veracity-labeled corrections alone and those following fake news reminders. To examine the sensitivity to detect this effect, we first calculated the odds ratio of the pairwise difference between these conditions. To do this, we modeled the effects of experimental manipulations in Experiment 1 on real news recall in Phase 3 using a logistic mixed effects model, fitted with the *glmer* function from *lme4* (Bates et al., 2015). We included the headline type predictor as a fixed effect and included by-participant and by-item random intercepts. We then conducted a significance test (z test) to derive the log odds ratio effect size for the difference between the conditions of interest using the *dotest* function from *simr* (Green & MacLeod, 2016). We converted the log odds ratio to an odds ratio using the *exp* function from *base R* and interpreted the effect size using the *interpret_oddsratio* function from the *effectsize* package (Ben-Shachar et al., 2020). The odds ratio for effect size of interest was small (*OR* = 1.46; Chen et al., 2010).

We conducted a simulation-based sensitivity analysis using *simr* (Green & MacLeod, 2016) to examine the power to detect the odds ratio from the comparison described above. A sensitivity analysis based on 1,000 simulations with alpha set at .05 revealed that with 97 participants, Experiment 1 had 98.30% [*95% CI* = 97.29, 99.01] power to detect a small effect (*OR* = 1.46). To further determine the sample size necessary to detect this effect we generated power curve showing power levels across varying sample sizes. The power curve below (see Figure S1) shows that based on 1,000 simulations with 80% power (alpha = .05), a sample size of approximately 43 participants was sufficient required to detect an *OR* = 1.46. Therefore, we decided that our sample size needed to be at least 43 participants. However, as we do not have prior data regarding how memory accuracy when fake news from phase 1 is re-activated by a partial or complete reminder, we have decided to remain conservative and choose a slightly larger sample size. According to the power curve (see Figure S1), a sample size of 60 exceeds the typical 80% power threshold to detect a small odds ratio (*OR* = 1.46).

**2. Qualitative and Quantitative Corrections**

The material set included both qualitative and quantitative corrections (See Figure 1). Qualitative corrections included changed sentence subjects. For example, the topic about the country ranked as having the cleanest air in 2019 included the fake news detail that it was the *United States*, and the real news detail that it was *New Zealand*. In contrast, quantitative corrections included changed amounts. For example, topic of the how much more the U.S women’s soccer team generated relative to the men’s included the fake news detail that it was *$20 million more* and the real news detail that it was *$5.8 million* more.

**3. Experiment 1 Sensitivity Analysis / Power Analysis for Experiments 2 and 3**

We conducted a simulation-based sensitivity analysis using R software (R Core Team, 2021) based on the results from Experiment 1, which also served as a power analysis for planning the sample size in Experiments 2 and 3. The smallest effect size of interest in Experiment 1 corresponded to the interaction between headline type and correct real news recall in Phase 3 conditioned on correction classification. To do this, we modeled the effects of experimental manipulations in Experiment 1 on real news recall in Phase 3 conditioned on correction classifications using a logistic mixed effects model, fitted with the *glmer* function from *lme4* (Bates et al., 2015). We included the headline type predictor as a fixed effect and included by-participant and by-item random intercepts. Two models were fit to the Experiment 1 data with different sample sizes, the first model contained 82 participants after we inaccurately removed 11 participants due to a response coding error; and the second model contained all 93 participants.

A sensitivity analysis based on 1,000 simulations with alpha set at .05 revealed that with 82 participants, Experiment 1 had 63.60% [*95% CI* = 60.53, 66.59] power to detect a Headline Type × Correction Classification interaction, χ^2^(2) = 6.74, *p* = .03. To further determine the sample size necessary to detect this interaction we generated power curve showing power levels across varying sample sizes. The power curve below (see Figure S2) shows that based on 1,000 simulations with 80% power (alpha = .05), a sample size of approximately 117 participants was sufficient required to detect an interaction effect.

A sensitivity analysis based on 1,000 simulations with alpha set at .05 revealed that with 93 participants, Experiment 1 had 55.10% [*95% CI* = 51.96, 58.21] power to detect a Headline Type × Correction Classification interaction, χ^2^(2) = 5.39, *p* = .07. To further determine the sample size necessary to detect this interaction we generated power curve showing power levels across varying sample sizes. The power curve below (see Figure S3) shows that based on 1,000 simulations with 80% power (alpha = .05), a sample size of approximately 154 participants was sufficient required to detect an interaction effect. Experiment 1 was thus under-powered for the smallest effect of interest, but Experiments 2 and 3 were sufficiently powered.

**References**

Bates, D., Mächler, M., Bolker, B., & Walker, S. (2015). Fitting linear mixed-effects models using lme4. *Journal of Statistical Software*, *67*(1). https://doi.org/10.18637/jss.v067.i01

Ben-Shachar, M., Lüdecke, D., & Makowski, D. (2020). effectsize: Estimation of effect size indices and standardized parameters. *Journal of Open Source Software*, *5*(56), 2815. https://doi.org/10.21105/joss.02815

Chen, H., Cohen, P., & Chen, S. (2010). How big is a big odds ratio? Interpreting the magnitudes of odds ratios in epidemiological studies. *Communications in Statistics - Simulation and Computation*, *39*(4), 860–864. https://doi.org/10.1080/03610911003650383

Green, P., & MacLeod, C. J. (2016). SIMR: An R package for power analysis of generalized linear mixed models by simulation. *Methods in Ecology and Evolution*, *7*(4), 493–498. https://doi.org/10.1111/2041-210X.12504

R Core Team. (2021). *R: A language and environment for statistical computing* [Computer software]. R Foundation for Statistical Computing. https://www.R-project.org/.

Wahlheim, C. N., Alexander, T. R., & Peske, C. D. (2020). Reminders of everyday misinformation statements can enhance memory for and beliefs in corrections of those statements in the short term. *Psychological Science*, *31*(10), 1325–1339. https://doi.org/10.1177/0956797620952797

**Figure S1**

**
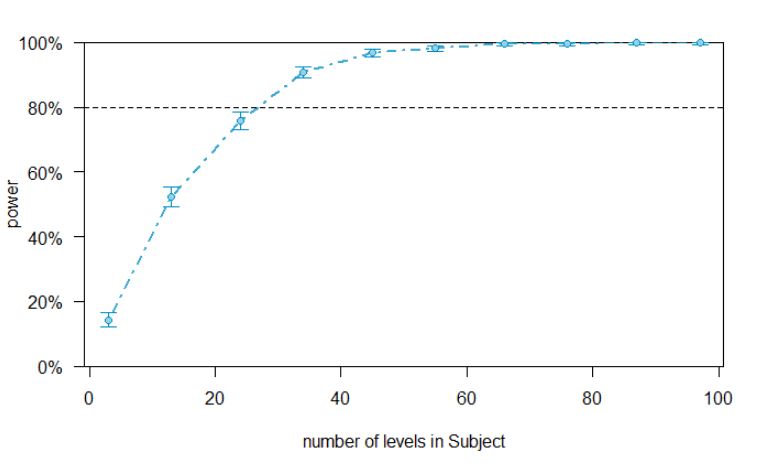
***Power Curve for Wahlheim et al. (2020; Experiment 1)*

*Note.* Power curve to detect a small effect (*OR* = 1.46) as a function of sample size (number of levels in Subject) using data from Wahlheim et al. (2020; Experiment 1).

**Figure S2**

*Power Curve for Experiment 1 with 82 participants*


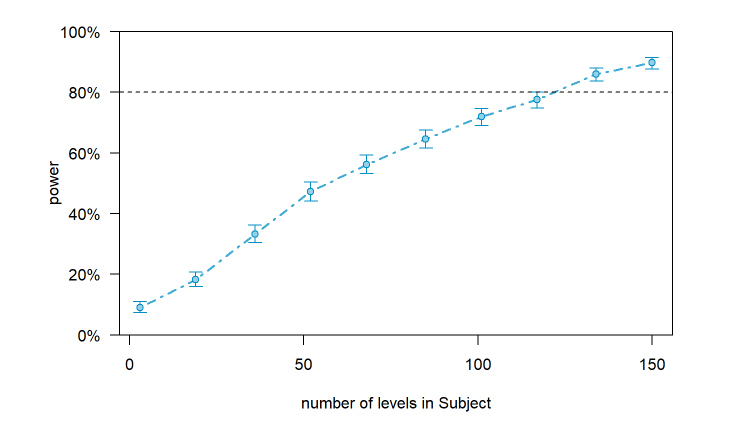


*Note.* Sensitivity curve to detect a Headline Type × Correction Classification interaction, χ^2^(2) = 6.74, *p* = .03 as a function of sample size (number of levels in Subject) using data from Experiment 1 (n = 82)

**Figure S3**

*Power Curve for Experiment 1 with 93 participants*

***
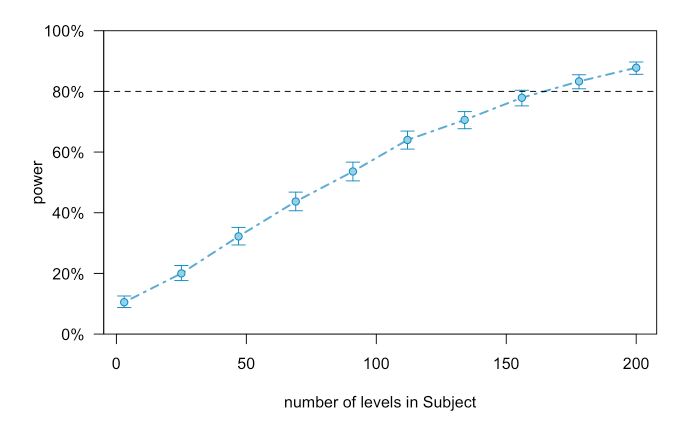
***

*Note.* Sensitivity curve to detect a Headline Type × Correction Classification interaction, χ^2^(2) = 5.39, *p* = .07, as a function of sample size (number of levels in Subject) using data from Experiment 1 (n = 93)
